# Exploring the antioxidant potential of prolactin

**DOI:** 10.3389/fphar.2025.1632087

**Published:** 2025-10-03

**Authors:** Funda Bulut Arikan, Nevin Sagsoz

**Affiliations:** ^1^ Department of Physiology, Faculty of Medicine, Kirikkale University, Kirikkale, Turkiye; ^2^ Department of Obstetrics and Gynecology, Faculty of Medicine, Kirikkale University, Kirikkale, Turkiye

**Keywords:** prolactin, total antioxidant status, total oxidant status, thiols, antioxidant, hormone

## Abstract

**Introduction:**

Prolactin (PRL) is primarily recognized for its role in lactation, yet its antioxidant function remains incompletely elucidated. The purpose of the present study was to examine the antioxidative role of prolactin.

**Methods:**

Seventy female volunteers were equally divided into hyperprolactinemia and control groups in this case–control study. Total antioxidant status (TAS), total oxidant status (TOS), native thiol (NT), total thiol (TT), prolactin, thyroid-stimulating hormone (TSH), free triiodothyronine (FT3), and free thyroxine (FT4) analyses were performed using the serum samples with the relevant kits based on the colorimetric or spectrophotometric methods. In addition, oxidative stress index (OSI), disulfide (DS), and the percent ratios of disulfide/native thiol (DS/NT), disulfide/total thiol (DS/TT), and native thiol/total thiol (NT/TT) were also determined.

**Results:**

TAS, NT, and TT concentrations and NT/TT percent ratio were found to be higher in patients than in the control group (*p* < 0.05). OSI and TOS levels, along with DS/NT and DS/TT percent ratios, were significantly lower in the hyperprolactinemia group than in the control group (*p* < 0.001 for all). FT4 was also lower in the hyperprolactinemia group (*p* < 0.05). Prolactin demonstrated a statistically significant positive correlation with TAS, NT, TT, and NT/TT and a significant negative correlation with TOS, OSI, and FT4 (*p* < 0.05). The TOS exhibits a statistically significant negative regression with prolactin, while NT has a statistically significant positive regression (*p* = 0.002 for both).

**Discussion/Conclusion:**

Hyperprolactinemia elevated total antioxidant and thiol levels while suppressing total oxidants, oxidative stress, and disulfide/thiol ratios. These findings indicate that prolactin plays a pivotal role in maintaining thiol–disulfide homeostasis and oxidative balance, functioning as a crucial endogenous antioxidant hormone. Moreover, the elevated prolactin levels during gestation and lactation may reflect its essential contribution to maternal–infant antioxidant defense, thereby supporting optimal fetal development.

## Graphical Abstract

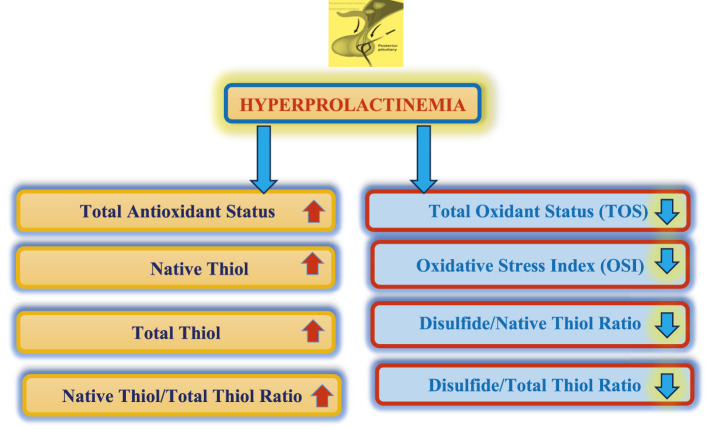

## Highlights

• This study stands out as the first to examine TAS, TOS, OSI, thiol levels, disulfide bond formation, and thiol/disulfide homeostasis in individuals with hyperprolactinemia.
• Hyperprolactinemia not only elevates levels of total antioxidants and thiols in the body but also reduces concentrations of total oxidants, oxidative stress, and disulfide/thiol percent ratios.
• Prolactin plays a crucial role in maintaining dynamic thiol–disulfide homeostasis and oxidative balance,
• Prolactin appears to function as a vital antioxidant hormone, revealing new dimensions to its physiological impact beyond traditional understandings.

## 1. Introduction

Prolactin (PRL) is a peptide hormone predominantly synthesized by lactotroph cells of the anterior pituitary under both physiological and pathological conditions (Melmed et al., 2011; Saba et al., 2025). Hyperprolactinemia, characterized by elevated serum PRL levels, commonly leads to amenorrhea, reproductive dysfunction, galactorrhea, and hypogonadism. Hyperprolactinemia may result from lactotroph adenomas (prolactinomas), which constitute approximately 40% of all pituitary tumors, but it may also occur secondary to disturbances in hypothalamic–pituitary dopaminergic regulation, primary hypothyroidism, chronic renal disease, structural hypothalamic–pituitary lesions, prolonged dopamine antagonist therapy, or idiopathic origin (Capozzi et al., 2015; Melmed et al., 2011; Colao, 2009).

Prolactin is involved in a wide range of biological functions, notably the regulation of reproductive processes and the initiation and maintenance of lactation (Capozzi et al., 2015; Melmed et al., 2011; Lopez-Vicchi et al., 2020). Aside from its many other known biological functions, emerging evidence suggests that prolactin may contribute to the reduction of cytosolic reactive oxygen species (ROS) and the maintenance of antioxidant capacity (Thébault, 2017). Despite these insights, the antioxidant function of prolactin remains insufficiently investigated in the current literature (Sies, 1997; Rice-Evans and Miller, 1994).

In biological systems, a delicate balance exists between antioxidant and oxidant molecules. Under certain circumstances, an increase in oxidants and a decrease in antioxidants become inevitable, shifting the oxidative/antioxidative balance toward an oxidative state (Sies, 1997; Rice-Evans and Miller, 1994; Forman and Zhang, 2021). Excessive ROS can inflict damage on cellular components such as mitochondria, nuclei, DNA, and membranes, contributing significantly to the pathogenesis of various diseases (Sies, 1997; Rice-Evans and Miller, 1994; Forman and Zhang, 2021; Buyukuslu and Yigitbasi, 2015; Erel, 2005). Antioxidants play a crucial role in mitigating the harmful effects of ROS, including superoxide anion radicals, hydrogen peroxide (H_2_O_2_), peroxyl radicals, and hydroxyl radicals (−OH), thereby maintaining the physiological balance (Sies, 1997).

Measurement of total antioxidant status (TAS) provides an assessment of the body’s overall antioxidant capacity, whereas total oxidant status (TOS) reflects the overall oxidant state. The oxidative stress index (OSI), ascertained as the ratio of TOS to TAS, provides insights into the oxidative/antioxidative balance within the body (Erel, 2005).

Moreover, thiols are crucial organic compounds with potent antioxidant properties, capable of trapping free radicals and forming reversible disulfide bonds (S–S) with oxidant molecules (Rice-Evans and Miller, 1994; Erel and Salim, 2014).

Albumin and protein thiols represent the major plasma thiol components, whereas low molecular weight thiols, including cysteinylglycine, cysteine (Cys), γ-glutamylcysteine, glutathione, and homocysteine, account for a smaller proportion. Thiols undergo oxidation by oxidant molecules, forming reversible disulfide bonds. These disulfide bond structures can be subsequently reduced back to thiol groups, thereby maintaining the thiol/disulfide balance. Maintaining a highly reduced intracellular environment under physiological conditions is vital, with thiols playing a pivotal role in thiol/disulfide balance regulation (Sies, 1997; Rice-Evans and Miller, 1994; Erel and Salim, 2014; Erel and Erdoğan, 2020).

Recently, studies have focused on oxidant/antioxidant molecules. Impairment of TOS/TAS and thiol/disulfide homeostasis takes part in the pathophysiology of diverse disorders, including diabetes mellitus, neurological diseases, cancer, cardiovascular diseases, renal failure, and liver diseases (Ates et al., 2015; Ayada et al., 2022; Das et al., 2024; Kar et al., 2021; Sogut et al., 2022; Terao et al., 2015; Uçmak and Yildizhan, 2025; Verma et al., 2016).

Therefore, elucidating these aspects of homeostasis may provide insights into the pathogenesis of various diseases and contribute to a better understanding of physiological functions. However, the specific mechanisms underlying the antioxidant activity of prolactin remain unclear; thus, to the best of our knowledge, the present study was designed as the first clinical human investigation to examine the antioxidative role of prolactin and its influence on oxidative parameters.

## 2. Methods

### 2.1 Subjects

This case–control study included 70 female participants recruited from the Department of Gynecology and Obstetrics at the University Medical Faculty Hospital. The volunteers were evenly divided into two groups: patients diagnosed with hyperprolactinemia and a control group, with an age range of 18–45 years. There were no significant differences in age or body mass index (BMI) between the hyperprolactinemia and control groups (*p* > 0.05). A *cut-off value* of 25 ng/mL was used for the serum PRL level to determine the hyperprolactinemia status of the patients (Melmed et al., 2011). Hyperprolactinemia was diagnosed only after confirmation by at least two repeat tests, and only those with persistent elevation were included in the study. The minimum–maximum values of prolactin levels for the patient group were 30–91.2 ng/mL.

#### 2.1.1 Exclusion criteria for both hyperprolactinemia and control groups

Participants who were using antipsychotics, antioxidant medications, or vitamin supplements, along with those who were pregnant, breastfeeding, or diagnosed with malignancy, pituitary adenoma, chronic kidney disease, chronic infectious diseases, or endocrine disorders (such as thyroid disease or diabetes mellitus), were excluded.

#### 2.1.2 Inclusion criteria for the hyperprolactinemia patient group

Participants had a serum PRL level above a *cut-off value* of 25 ng/mL and did not meet any exclusion criteria.

#### 2.1.3 Inclusion criteria for the control group

Participants had a serum PRL level less than 25 ng/mL and did not meet any exclusion criteria.

### 2.2 Ethics statement

The study was approved by the Clinical Research Ethics Committee of the Faculty of Medicine, Kirikkale University. Volunteers received detailed information about the study and voluntarily signed the informed consent form.

### 2.3 Study protocol

Demographic characteristics of the volunteers, including age, height, and weight, were recorded. BMI was calculated as [weight (kg)/height (m)^2^]. Blood samples were collected from both patients and controls in the morning after 8 hours of fasting, and approximately 10 mL of venous blood was obtained from each participant. Blood samples collected on days 2–3 of menstruation, during the follicular phase, were centrifuged at 3,500 rpm for 10 min to obtain serum within 30 min of collection. The serum samples were then stored at −80 °C until further analysis, including the measurement of TAS, TOS, native thiol (NT), total thiol (TT), prolactin, thyroid-stimulating hormone (TSH), free triiodothyronine (FT3), and free thyroxine (FT4) concentrations. Additionally, OSI, disulfide (DS), and the ratios of disulfide/native thiol (DS/NT), disulfide/total thiol (DS/TT), and native thiol/total thiol (NT/TT) percentages were also analyzed in this study.

For the detection of TAS, TOS, NT, and TT concentrations, RelAssay^®^ Diagnostics kits (Mega Medicine) were used, which are based on colorimetric or spectrophotometric methods as described by Erel (2005) and Erel and Salim (2014). The TAS test kit is based on the bleaching method, using antioxidants to reduce 2,2′-azinobis-(3-ethylbenzothiazoline-6-sulfonic acid) radical cation (ABTS*+), which is stable and colorful. The measurement principle involves detecting this color change using a spectrophotometer (Erel, 2004). TOS levels were determined using a colorimetric kit based on the oxidation of ferrous ion to ferric (Erel, 2005). TAS and TOS kits measured absorbance at wavelengths of 660 nm and 530 nm, respectively. Native and total thiol concentrations were measured using spectrophotometric kits developed by Erel and Neselıoglu, modified from the Ellman method, with measurements taken at a wavelength of 415 nm (Erel and Salim, 2014). The OSI was computed as the percent ratio of TOS to TAS levels. Half of the difference between TT and NT concentrations was considered the level of disulfide (Erel and Salim, 2014). The change in absorbance at 660 nm is proportional to the TAS level of the sample. The ferrous ion–chelator complex is oxidized to ferric ion by oxidants in the serum sample. In acidic media, ferric ions form a colorful complex with xylenol orange. The change in absorbance at 530 nm is directly proportional to the sample’s TOS level.

Prolactin (PRL II assay), TSH, FT3, and FT4 serum concentrations were detected using Roche Elecsys kits with the electrochemiluminescence immunoassay technique on the Roche cobas e801 analyzer (Roche Diagnostics GmbH, Mannheim, Germany). Analytical performance characteristics of ELISA kits are demonstrated in Table 1.

**TABLE 1:** Comparison of demographic and clinical data.

Parameters	Control n = 35	Hyperprolactinemia n = 35	p
Age	26.6 ± 7.3	29.1 ± 7.6	0.17
BMI	22.5 ± 2	24 ± 4.4	0.09
Prolactin (ng/mL)	14.5 ± 5.9	52.8 ± 20.9	<0.001**
TAS (mmol Trolox eq/L)	1.02 ± 0.2	1.13 ± 0.2	0.014*
TOS (µmol H_2_O_2_ eq/L)	21.48 ± 11	12.38 ± 5.2	<0.001**
OSI (arbitrary units)	2.23 ± 1.4	1.12 ± 0.48	<0.001**
Native thiol (µmol/L)	166.47 ± 36.5	207.65 ± 41	<0.001**
Total thiol (µmol/L)	261.43 ± 39.2	292.4 ± 49.1	0.006*
Disulphide (µmol/L)	49.5 ± 22.8	42.3 ± 24	0.21
Disulphide/native thiol %	32.7 ± 18.9	22.4 ± 15.3	0.016*
Disulphide/total thiol %	18.33 ± 6.6	14.01 ± 7.1	0.012*
Native thiol/total thiol %	78.8 ± 17.3	98.3 ± 19.4	<0.001**
TSH (uIU/mL)	1.98 ± 1	2.07 ± 0.9	0.710
FT3 (pg/mL)	3.1 ± 0.3	3.02 ± 0.4	0.430
FT4 (ng/dL)	1.28 ± 0.1	1.20 ± 0.1	0.021*

TAS, total antioxidant status; TOS, total oxidant status; OSI, oxidative stress index; TSH, thyroid-stimulating hormone; FT3, free triiodothyronine; FT4, free thyroxine. Data shown are as the mean ± SD.

*p < 0.05 and **p < 0.001.

### 2.4 Statistical analysis

The statistical analyses were conducted using SPSS, version 27.0 (IBM SPSS Statistics, Armonk, NY, United States), and the figures were generated using Python (version 3.10). Power analysis was carried out to determine the required sample size for a 0.80 power and a 0.05 significance level. The distribution of the data was assessed separately for the control and hyperprolactinemia groups using the Shapiro–Wilk test. Age, BMI, prolactin level, and TSH level did not follow a normal distribution; therefore, the Mann–Whitney U test was used for the comparison of these parameters. In contrast, the remaining variables exhibited a normal distribution and were analyzed using the Student’s t-test. Correlations between variables were evaluated using Pearson’s correlation test for normally distributed data and Spearman’s rank correlation test for non-normally distributed data. Statistical significance was set at *p* < 0.05. Furthermore, multiple regression analyses were performed to assess the effects of TAS (mmol Trolox eq/L), TOS (µmol H2O2 eq/L), NT (µmol/L), and TT (µmol/L) levels on serum prolactin concentrations.

## 3 Results

Comparison of demographic characteristics and laboratory data for the groups is presented in Table 2. TOS levels, OSI values, and DS/NT and DS/TT percent ratios were significantly lower in the hyperprolactinemia group than in the control group (p < 0.001 for all). Although the mean serum level of FT4 remains within the normal laboratory range (0.93–1.7 ng/dL), it was observed to be lower in the hyperprolactinemia group.

**TABLE 2:** Analytical performance characteristics of ELISA kits.

Hormone	Intra-assay CV (%)	Inter-assay CV (%)	Measuring range	Limit of detection
Prolactin	1.8–3.1	2.6–4.4	2–10,000 μIU/mL (0.094–470 ng/mL)	20 μIU/mL (0.940 ng/mL)
Free T3	1.4–7.6	1.6–8.3	0.6–50 pmol/L	1.5 pmol/L
Free T4	1.5–5.3	3.2–14.0	0.5–100 pmol/L	1.3 pmol/L
TSH	0.7–3.4	1.5–11.2	0.005–100 μIU/mL	0.005 μIU/mL

Prolactin, TAS, NT, and TT concentrations and the NT/TT percent ratio were found to be higher in the hyperprolactinemia group than in the control group (*p* < 0.001, Table 2).

**FIGURE 1:**
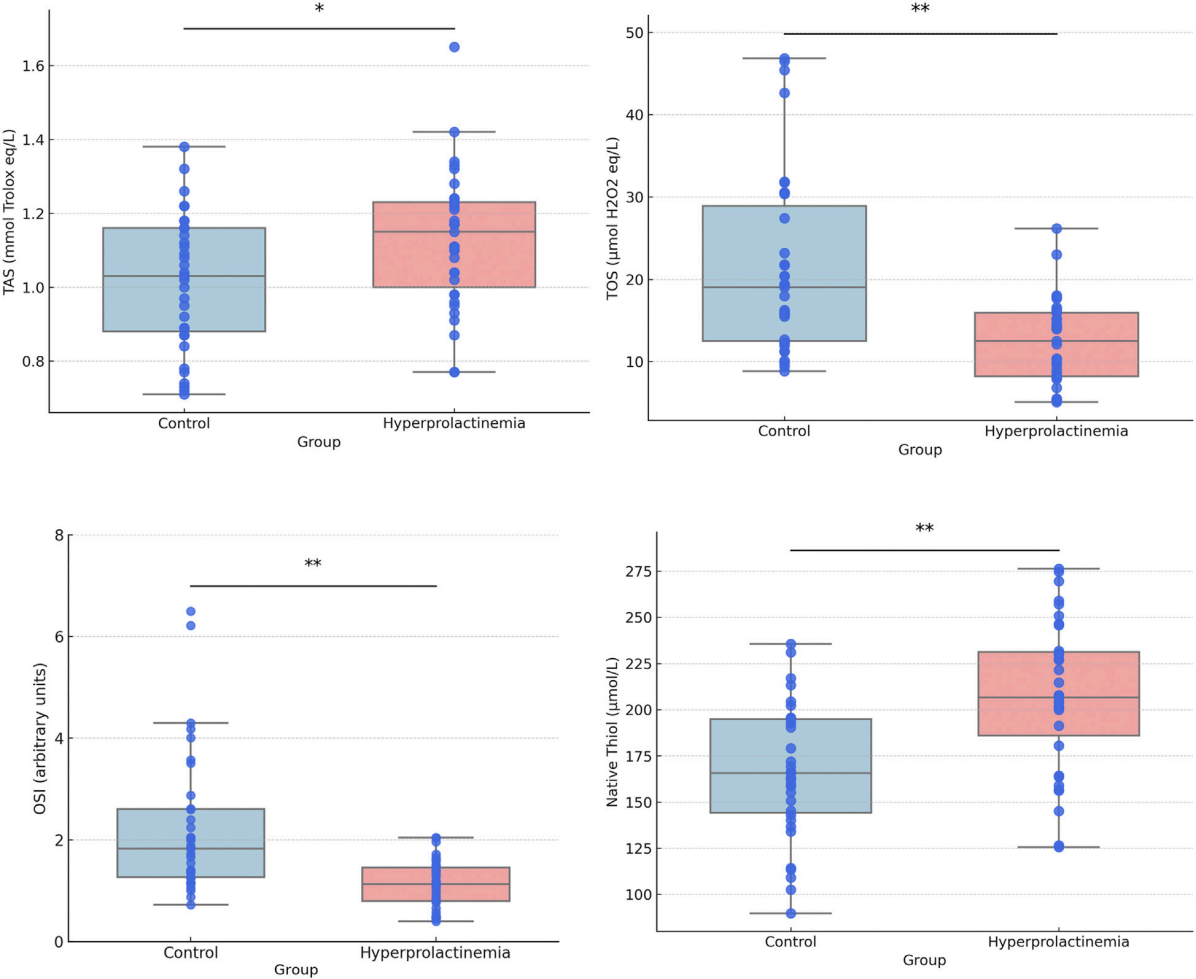
Box- and whisker plots with individual data points for TAS, TOS, OSI, and native thiol. Each plot displays the median and the interquartile range (25th–75th percentiles, lower and upper bounds).

**FIGURE 2:**
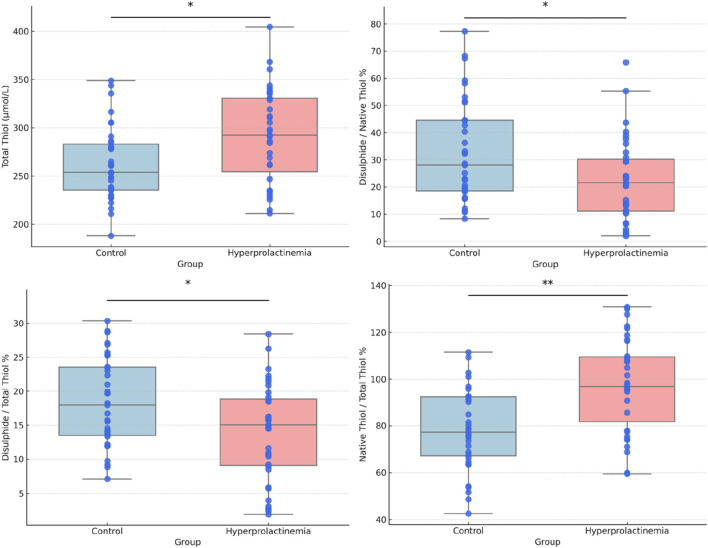
Box and whisker plots with data points for total thiol and the ratios of native thiol/total thiol, disulphide/native thiol, and disulphide/total thiol. Each plot shows the median and the interquartile range (25th–75th percentiles).

**FIGURE 3:**
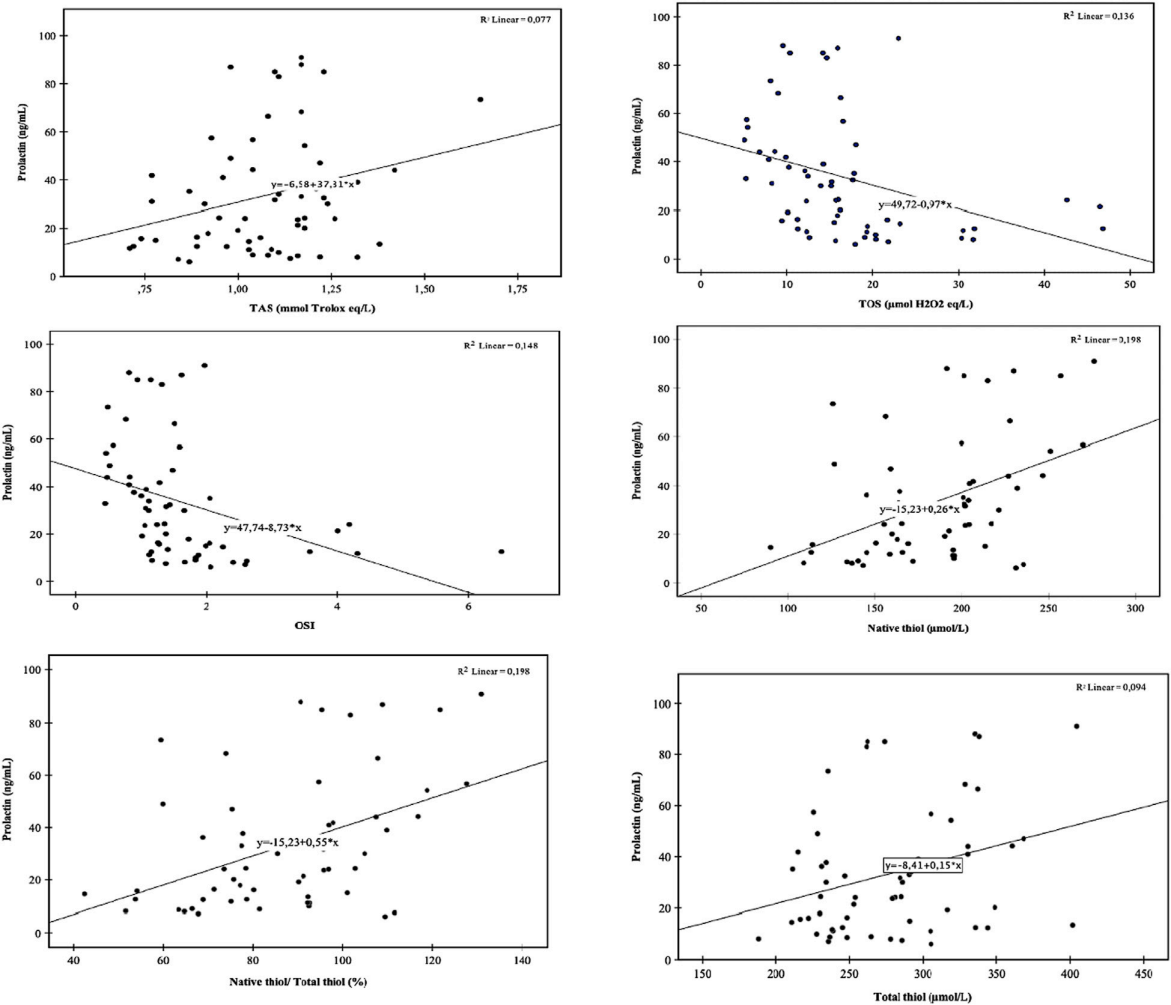
Correlation analyses. TAS, total antioxidant status; TOS, total oxidant status; OSI, oxidative stress index.

Box and whisker plots for comparing TAS, TOS, OSI, native and total thiol concentrations, and percent ratios of NT/TT, DS/NT, and DS/TT are presented in Figures 1, 2. Correlation analyses were also conducted, and the results are provided in Figure 3. Serum prolactin concentrations exhibited a statistically significant positive correlation with TAS, TT, NT, and NT/TT, while a statistically significant negative correlation was found with TOS and OSI levels in all groups (*p* < 0.05). A statistically significant negative correlation was also found between prolactin and FT4 (r = −0.361; *p* = 0,016).

Based on the analysis, a significant regression model was observed [F (4, 63) = 9.94; *p* < 0.001], explaining that 35% of the variance in the dependent variable was accounted for by the independent variables (*R* ^2^ adjusted = 0.35). According to the results of the regression analyses, TOS has a statistically significant negative effect on prolactin levels [β = −0.34, t (63) = −3.27, *p* = 0.002, and pr^2^ = 0.14], while NT levels have a statistically significant positive effect, i.e., β = 0.37, t (63) = 3.16, *p* = 0.002, and pr^2^ = 0.14. The regression equation is as follows: 0.44 − 0.019 × TOS + 0.004 × NT.

**FIGURE 4:**
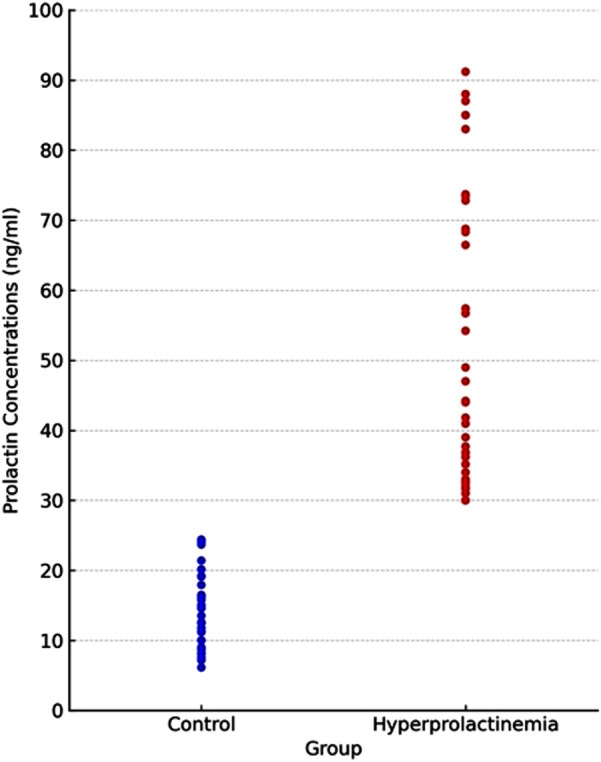
Distribution of prolactin levels between groups.

The distribution of prolactin concentrations is shown in Figure 4.

## 4 Discussion

The findings of the present study demonstrated a shift toward the antioxidant side in hyperprolactinemia, suggesting that prolactin functions as a potent antioxidant hormone. To the best of our knowledge, this study is the first to demonstrate that hyperprolactinemia increases TAS, NT, and TT antioxidant concentrations, along with NT/TT percent ratios, while decreasing OSI values, TOS levels, and DS/NT and DS/TT percent ratios.

There is a paucity of literature exploring the antioxidant effects of prolactin. Data from the present study are consistent with findings from cell culture studies conducted by Rivero-Segura et al. (2019) and García et al. (2016). Segura et al. investigated primary cultures of hippocampal neurons and demonstrated that prolactin enhances the activity of superoxide dismutase (SOD), a key antioxidant enzyme counteracting oxidative stress, while increasing the protein content of both isoforms (Mn^2+^–SOD and Cu^2+^/Zn^2+^–SOD) under conditions of glutamate-induced excitotoxicity. In addition, prolactin markedly reduced lipid peroxidation (LPO) products such as malondialdehyde (MDA), which are generated in response to excessive ROS production. Notably, MDA is widely recognized as a reliable biomarker of oxidative stress (Rivero-Segura et al., 2019). However, in contrast to the findings of Segura et al., a study on retinal pigment epithelium (RPE) cell lines by García et al. reported that prolactin neither modifies the H_2_O_2_-induced increases in Mn^2+^–SOD, Cu^2+^/Zn^2+^–SOD, and glutathione peroxidase (GPx) concentrations nor affect catalase mRNA expression. Nonetheless, García et al. demonstrated that prolactin enhanced the antioxidant capacity of RPE cells by reducing glutathione levels and inhibiting the H_2_O_2_-mediated upregulation of the deacetylase sirtuin 2 (SIRT2), which is associated with increased susceptibility to cell death under oxidative stress. Furthermore, they showed that the activation of the PRL receptor conferred resistance to oxidative stress in RPE cells, whereas oxidative stress was exacerbated in mice lacking the prolactin receptor (PRLR^−/−^) (García et al., 2016). In another cell culture study, Yamamoto et al. demonstrated that administration of recombinant human prolactin (rhPRL) to pancreatic β-islet cell cultures promoted β-cell survival and conferred protection against cytotoxic agents such as H_2_O_2_ and nitric oxide donors *in vitro*. Based on these findings, they proposed that PRL supplementation prior to transplantation may enhance islet graft success by minimizing β-cell loss (Yamamoto et al., 2010). However, a cell culture study reported that prolactin modulates the oxidative/antioxidative balance in a dose-dependent manner. In ovine ovarian granulosa cells, high PRL concentrations downregulated PRL receptors and induced oxidative stress-mediated apoptosis (Yang et al., 2023). The underlying reasons for these discrepancies remain unclear but may be related to differences in cell types and their respective metabolic characteristics examined across studies. Moreover, the antioxidant activity of prolactin may vary depending on the specific cell type or circulating hormone concentrations.

Although these findings offer important mechanistic insights at the cellular level, they require validation in clinical settings. The current study is the first clinical investigation to demonstrate that hyperprolactinemia enhances antioxidant capacity while reducing oxidant burden. Systemic redox balance was comprehensively evaluated using TAS, TOS, OSI, and thiol–disulfide homeostasis. TOS reflects the cumulative oxidative burden, whereas TAS represents the overall antioxidant capacity. OSI provides an index of the oxidant/antioxidant equilibrium, and thiol–disulfide parameters offer direct insight into protein oxidation and redox homeostasis (Erel, 2004; Erel and Erdoğan, 2020; Erel, 2005; Erel and Salim, 2014). Collectively, these markers enabled a comprehensive evaluation of oxidative stress, providing an integrated perspective beyond individual enzymatic measurements. In line with the results of our study, a previous study on patients with psychotic disorders treated with risperidone or paliperidone reported that female participants exhibited higher prolactin levels and a greater prevalence of hyperprolactinemia. Antipsychotic therapy significantly influenced oxidative parameters, and prolactin was inversely correlated with TBARS, O_2_^−^, and SOD, suggesting a protective role against oxidative stress (Stojkovic et al., 2024).

In light of previous studies, the physiological mechanisms by which prolactin modulates oxidant and antioxidant systems remain unclear (Forman and Zhang 2021). Recent evidence has suggested that prolactin may exert antioxidant effects through specific intracellular signaling pathways. Prolactin interacts with its receptor to activate the Janus kinase 2 (JAK2)-signal transducer and activator of transcription 5 (STAT5) signaling cascade, a pathway that plays a pivotal role in regulating cellular survival and proliferation (Molina-Salinas et al., 2021; Zhang et al., 2019; Ulloa et al., 2024). Mechanistically, STAT5 activation induces nuclear translocation and regulates target genes, including antioxidant enzymes such as SOD and glutathione peroxidase. In addition to STAT5, prolactin signaling can also activate STAT3 and cross-talk with NRF2, thereby inducing the expression of antioxidant response elements such as SOD1 and SOD2 (Molina-Salinas et al., 2021; Ulloa et al., 2024; Rivero-Segura et al., 2019).

These findings collectively indicate that the antioxidant effect of prolactin is underpinned by the interplay of multiple signaling pathways. In this context, the PI3K/AKT–NF-κB axis may provide a direct contribution by enhancing SOD1 and SOD2 expressions, while the JAK2/STAT5 and AKT–GSK3β/FOXO pathways offer additional antioxidant support. The MAPK/ERK1/2 pathway, although primarily associated with proliferation, may provide secondary contributions to antioxidant capacity.

Through the coordinated actions of these signaling cascades, prolactin may enhance redox homeostasis and mitigate oxidative stress (Molina-Salinas et al., 2021; Ulloa et al., 2024; Rivero-Segura et al., 2019; Zhang et al., 2019). Consistent with these mechanistic insights, the findings of this study, demonstrating increased antioxidant levels and preservation of the thiol pool in hyperprolactinemia, further support the hypothesis that prolactin functions as an endogenous antioxidant hormone, thereby providing protection against oxidative stress at the clinical level. The mechanistic framework linking prolactin to antioxidant defense may also provide an explanation for the thiol–disulfide findings of our study. By limiting the generation of ROS and enhancing their clearance, prolactin signaling may reduce the oxidation of thiol groups into disulfide bonds. Furthermore, the upregulation of GPx and related enzymes may strengthen the glutathione redox system, thereby contributing to the preservation of the thiol pool.

Moreover, NF-κB- and Bcl-2-mediated maintenance of mitochondrial integrity prevents excessive ROS formation, which, in turn, helps limit disulfide accumulation (Susnow et al., 2009; Zhang et al., 2019; Ulloa et al., 2024). These mechanisms together indicate that prolactin, through coordinated activation of the JAK2/STAT5, STAT3, NRF2, and PI3K/AKT pathways, may facilitate a shift toward a more favorable thiol–disulfide equilibrium, aligning with the decreased disulfide ratios observed in our hyperprolactinemia cohort.

By engaging potential signaling pathways, prolactin protects diverse cell types, including pancreatic β-cells, hippocampal neurons, and retinal pigment epithelial cells, against apoptosis and exerts antioxidant effects (Rivero-Segura et al., 2019; García et al., 2016; Yamamoto et al., 2010). This protective effect of prolactin is likely to extend to other tissues where the prolactin receptor is expressed, including the mammary gland, pituitary, ovary, uterus, kidney, liver, and immune cells (Peirce et al., 2001; Nagano and Kelly, 1994). Therefore, the physiological increase in prolactin concentrations during pregnancy and breastfeeding may provide very important antioxidant protection for both mother and baby.

Furthermore, in the present study, patients with hyperprolactinemia were considered feasible for cross-sectional observation of whether prolactin exerts an antioxidative effect in humans as alternative study designs are ethically impractical.

The findings of the present study should be interpreted with due regard to its methodological constraints. The case–control design inherently precludes definitive causal inferences, and the restriction to a female-only cohort, together with the modest sample size, limits the external validity and generalizability of the conclusions. In addition, the absence of mechanistic assays constrains the depth of biological insight attainable. Accordingly, future large-scale, mechanistically oriented investigations are warranted to substantiate and extend these observations.

## 5 Conclusion

The current study provides the first clinical evidence that hyperprolactinemia enhances systemic antioxidant and thiol levels while suppressing oxidants, oxidative stress, and disulfide/thiol ratios. Prolactin thereby emerges as a pivotal regulator of thiol–disulfide homeostasis, extending its role beyond lactation and reproduction to encompass antioxidant protection. Nevertheless, confirmation through rigorously designed clinical and mechanistic studies is warranted.

## Data Availability

The raw data supporting the conclusions of this article will be made available by the authors, without undue reservation.
